# Prevalence and risk factors of acute lower respiratory infection among children living in biomass fuel using households: a community-based cross-sectional study in Northwest Ethiopia

**DOI:** 10.1186/s12889-020-08515-w

**Published:** 2020-03-19

**Authors:** Mesafint Molla Adane, Getu Degu Alene, Seid Tiku Mereta, Kristina L. Wanyonyi

**Affiliations:** 1 Department of Environmental Health, School of Public Health, College of Medicine & Health Sciences, Bahir Dar University, Bahir Dar, Ethiopia; 2 Department of Epidemiology and Biostatistics, School of Public Health, College of Medicine & Health Sciences, Bahir Dar University, Bahir Dar, Ethiopia; 3 Departments of Environmental Health Science and Technology, Jimma University, Jimma, Ethiopia; 4 Department of Dental Academy, Faculty of Science, University of Portsmouth, Portsmouth, England

**Keywords:** Baking stove, Childhood ARI, Chimney, Cooking fuel, Cookstove, Indoor burning

## Abstract

**Background:**

Childhood acute lower respiratory infection in the form of pneumonia is recognized as the single largest cause of childhood death globally accounting for 16% of the overall deaths. Some studies also reported a higher prevalence of childhood acute respiratory infection in Ethiopia, which ranges from 16% up to 33.5%. Concerning the risk factors, there are limited community-based studies in Ethiopia in general, and in the current study region in particular. Therefore, the present study was conducted to investigate the prevalence of childhood acute respiratory infection and associated factors in Northwest Ethiopia.

**Methods:**

As part of the wider stove trial project, a cross-sectional study was conducted in May 2018 among a total of 5830 children aged less than 4 years old in randomly selected clusters. Binary logistic regression was applied to identify factors linked with childhood acute lower respiratory infection and adjusted odds ratios were used as measures of effect with a 95% confidence interval.

**Results:**

A total of 5830 children were included in the study within 100 clusters. Out of which 51.7% were male and 48.3% female. The prevalence of childhood lower acute respiratory infection was 19.2% (95% CI: 18.2–20.2) and found to decrease among children living in homes with chimney, eaves space and improved cookstove than children living in households with no chimney, eaves space and improved cookstove with estimated AOR of 0.60 (95% CI: 0.51–0.70), 0.70 (95% CI: 0.60–0.84) and 0.43 (95% CI: 0.28–0.67) respectively. It was also associated with other cooking-related factors such as cow dung fuel use [AOR = 1.54 (95% CI: 1.02–2.33)], child spending time near stove during cooking [AOR = 1.41 (95% CI: 1.06–1.88), presence of extra indoor burning events [AOR = 2.19 (95% CI: 1.41–3.40)] and with frequent cooking of meals [AOR = 1.55 (95% CI: 1.13–2.13)].

**Conclusion:**

High prevalence of childhood acute lower respiratory infection was demonstrated by this study and it was found to be associated with household ventilation, cooking technology, and behavioral factors. Therefore, we recommend a transition in household ventilation, cooking technologies as well as in child handling and in the peculiar local extra indoor burning practices.

## Background

Acute respiratory infection (ARI) is the leading cause of morbidity and mortality in low- and middle-income countries (LMICs) [1] across all ages and sexes [2]. Lower respiratory infections caused 652, 572 deaths in children younger than 5 years worldwide in 2016 [3]. Particularly, acute lower respiratory infection (ALRI) in the form of pneumonia is recognized as the single largest cause of childhood death globally accounting for 16% of the overall deaths in 2015 [4].

In Ethiopia, lower respiratory infections were the leading cause of premature mortality across all ages in the year 2015 [5] and pneumonia, in particular, was the second most common cause of mortality in Northeast Ethiopia [6]. In addition, the 2016 EDHS report confirmed a countrywide ARI prevalence of 7% among children under 5 years old [7], and a number of cross-sectional studies conducted in different parts of the country reported an even higher prevalence of childhood ARI in Ethiopia which ranges from 16% up to 33.5% [8–12].

Concerning the risk factors, studies have shown that household air pollution (HAP) exposure is linked with childhood ALRI [13, 14]. However, the factors are not identical across different circumstances and further investigations should be extended to other locations, populations, and cultures to determine the relationships more precisely [15]. There are, however, limited community-based studies in Ethiopia in general [16–18], and there is a robust lack of evidence in the current study region in particular as reported by a recent synthesis of situational analysis in Ethiopia [16]. The availability of such evidence would drive commitments by governments, industries, non-governmental organizations, communities, and individual citizens to find suitable HAP prevention methods in order to reduce the burden of preventable HAP related child ARI morbidity and mortality in Ethiopia and somewhere else in LMICs.

Therefore, taking account of the critical need for more evidence in the area as recommended by previous studies [16–18], the present study was conducted to assess the prevalence and factors associated with community-acquired childhood ALRI using the definition of Integrated Management of Childhood Illnesses (IMCI) pneumonia algorithm developed by World Health Organization (WHO) [19, 20]. The IMCI-pneumonia assessment protocol is a common diagnostic criterion for the classification and treatment of childhood pneumonia at health facilities in Ethiopia [21]. The Ethiopian Demographic and Health Survey (EDHS) [7] and other similar studies conducted in different parts of the world also used this diagnostic criterion for childhood pneumonia assessment; such as the randomized controlled trials conducted in Malawi [22], Rwanda [23], Ghana [24], Indian [25] and Guatemala [26]. In this study, we used the term childhood ALRI as a synonym for the IMCI-pneumonia, because the term childhood ALRI is the preferred expression for childhood pneumonia in peculiar to the developing world countries [27].

## Methods

### Study design

As part of the wider stove trial project in Northwest Ethiopia (ClinicalTrials.gov Identifier: NCT03612362), a community-based cross-sectional study was employed to determine the prevalence of childhood ALRI & associated factors among children living in biomass fuel using households in Northwest Ethiopia in which biomass fuel use is a major household energy source for cooking.

### Study setting

This study had been conducted in a low-income rural community of the Mecha Health and Demographic Surveillance System (MHDSS) site in which biomass fuel use is a major household energy source for cooking. MHDSS site is a field research center established in 2013 by Bahir Dar University to conduct and support postgraduate level studies in the region. It is located 525 km away from the capital city of Ethiopia, Addis Ababa, towards Northwest and 40 km far away from the capital city of Amhara Regional State, Bahir Dar. The study area comprises three major climatic zones of highlands (“Dega”), Midlands (“Weina Dega”), and lowlands (“Kola”). It also consists of 10 randomly selected Kebeles (sub-districts / the smallest administrative unit) of three urban and 7 rural with a total of 132 clusters/ “*Gots*”. According to the official population profile report of MHDSS, a total of 65, 086 population was registered within 20, 631 households at the end of 2016. Out of which children 0–4 years old were accounted for 13.3% of the total population.

### Study population

Since this study was part of the wider stove trial project, all households with children aged less than 4 years old with respective mothers/caretakers were recruited in the selected clusters to ensure a minimum of one-year longitudinal data collection before the child’s 5th birthday.

### Sample size determination

The sample size of this study was calculated using the population survey formula on EPI INFO program assuming a 95% confidence level, a 3% acceptable margin of error, 21% acceptable estimated population proportion of child ARI in Ethiopia from a previous study [10], a design effect of 2 for cluster sampling, allowing a 5% addition to account for any unpredictable events and assuming an average number of eligible children within each cluster to be 55 from the updated data of MHDSS, the final estimated sample size was about 1490 eligible children within 27 randomly selected clusters.

However, since we had the advantage of using all the baseline data of the wider stove trial project (ClinicalTrials.gov Identifier: NCT03612362), we considered all the randomly selected 100 clusters containing about 5830 eligible children from the stove trial project in order to attain the advantage of having a larger sample size which would reduce the sampling error thereby increasing the accuracy of estimations. We were also aware of the possible bias that might be introduced, and we did everything possible to deal with it.

### Participant sampling technique

The cluster sampling method was used to select participants using the small villages termed as “*Got*” in the local language as cluster units. Among the total 132 clusters in the MHDSS site, 100 clusters were selected randomly to represent the total population and all eligible households were included within the selected clusters. The sampling frame, list of households with children aged less than 4 years, was established from the MHDSS record, and the selected households were identified using the permanent MHDSS site house number. Then, the youngest child less than 4 years old was recruited from each household. In situations where there were two or more children aged under-4 years living in the same household, only the youngest child was included in the study.

### Variables, data sources, and methods of assessment

Outcome variable: To classify community-acquired childhood ALRI status (Yes or No), this study used the definition of WHO IMCI-pneumonia [19, 20]. Childhood ALRI was assessed by field nurses through face-to-face interviews where mothers/caregivers of the index children were asked about childhood ALRI/Pneumonia signs and symptoms in the preceding 2 weeks. Besides, since nurses trained in IMCI can effectively diagnose ALRI/pneumonia in Ethiopia [21]; physical examination of the ill index child was also conducted by the field nurses using the IMCI pneumonia algorism to overcome some of the limitations of the self-reported health data.

Predictor variables: The predictor variables were categorized into socio-demographic, ventilation, technology, and behavioral as well as other potential predictor variables. These variables were assessed through face-to-face interviews with index child mothers as well as through observations by field workers using questionnaire as mentioned next:

Ventilation: Four features of household ventilation were assessed:

- Chimney was assessed by asking and observing the existence of chimney in the main cooking quarter by classifying into two categories as Yes or No.
- Eaves space was assessed by asking and observing the presence of an open eaves space between the wall and the roof of the main cooking quarter by classifying into two categories as Yes or No.
- The number of windows was assessed through observing the number of windows in the main baking/ cooking area if any.
- Roof leak was assessed through asking and observing the presence of roof leak in the main cooking quarter by classifying into two categories as Yes or No.

Technology: Three aspects of household cooking technology-related factors were considered:

- “Injera” baking stove type was assessed by asking about and observing the primary type of stove used for “Injera” baking purpose and classifying these into two categories i] improved “Injera” baking stove (IBS) ii] traditional baking stove (TBS). The term IBS refers to energy-efficient baking stoves produced by certified manufactures and on sale at commercial markets in Ethiopia and TBS refers to baking stoves constructed by household members for “Injera” baking purpose, which happen to lack energy efficiency and have poor combustion features.
- Cookstove type was assessed by asking and observing the primary cooking stove type by classifying into two categories as improved cookstove or traditional cookstove (TCS). The term ICS refers to a household cooking stove that is designed to use less fuel, save time and reduce the volume of smoke produced compared to the traditional stove and the term traditional cookstove refers to a cookstove constructed by household members for cooking purpose that are not energy-efficient and have poor combustion features [12].
- Fuel type refers to the source of energy used for household cooking [12, 28], and since all study households primarily use biomass fuel, it was assessed through asking & observing the primary fuel type used for household cooking purposes by classifying into 3 categories as wood/straw/shrub, cow dung, and charcoal.

Behavioral: The following are the five behavioral factors investigated in this study:

- Child near the stove (child handling practice) was assessed by asking mothers whether their child regularly spent time near stove within 1.5-m distance during cooking times by classifying into two categories as Yes or No.
- Cooking time was assessed through asking respondents what the average time taken for cooking was in hours per day and classifying these into three categories as i] 1–2 h, ii] 3–4 h and iii] 5 or more hours per day.
- Extra indoor burning event was also assessed. This refers to any extra burning event (such as the burning of incense, coffee ceremony, local alcohol/ “*areqi*” making or cooking for business) that commonly occurs inside the main cooking quarter other than the customary cooking and may influence the HAP level as alternative sources of HAP. It was assessed by asking and observing the presence of any extra indoor burning occasion inside a household by classifying into two categories (Yes or No).
- Frequency of meal was assessed through asking the number of meals cooked per day by classifying into 4 categories as one or less meal, two, three and four/more meals
- Window opening practice of the households was assessed by asking the respondent and observing whether the windows were commonly opened in the main cooking quarter.

Other variables: Other variables were also assessed through face-to-face interviews with index child mothers as well as through observations by field workers as mentioned next

- Breast-feeding was assessed by asking mothers whether the child was exclusively breastfed for the first 6 months by classifying into two categories (Yes or No) [29, 30].
- Child vaccination refers to the immunization status of the child for his/her respective age by classifying into two categories (Yes/No) & assessed through asking/card checking
- An outdoor burning event refers to the presence of any extra burning event (such as burning rubbish, charcoal production or local alcohol/“*areqi*” making that commonly occurs nearby the household and may influence the HAP as an alternative source of HAP exposure. It was assessed by asking & observing the presence of any outdoor burning event near the household by classifying into two categories (Yes or No).
- Secondary smoking refers to cigarette smoking by a member of the household and it was assessed by asking the presence of any smoking practice at home by classifying into two categories (Yes or No).

### Data collection

Data collection was carried out in May 2018 by trained local nurses through face-to-face interviews with the mothers/caregivers of index children using a pre-tested questionnaire as well as through direct verification (i.e. examination of the ill child) using the IMCI algorithm [19]. The data collection questionnaire (Provided in additional file 1) was developed after a comprehensive literature review and expert evaluation based on the specific objectives of the study. It contains household location, socio-demographic, index child health, main cooking quarter characteristics, cooking pattern and alternative sources of household air pollution related questions of the study households.

To prevent missing data and avoid the associated complexities in data analysis and interpretation, several special efforts were applied before and during the data collection period. To mention some; the stove trial research project was publicized through a live discussion on the Regional Television show in May 2018 to build community interest in the study. Interest in the study was also created through communications about the study during the regular local health development army team meetings as well as through home visits by the local health and agricultural extension workers along with local energy experts by means of working on active community engagement through the Ethiopian health and agricultural extension programs as well as through the local health development army team structure.

Participants were also informed that they will receive an IBS for free either at the initiation or at the end of the study to maintain justice and to help achieve a high level of research participation. Besides, we utilized local household energy experts and health extension workers to oversee the overall efforts in recruiting eligible households. Furthermore, home visits for data collection were scheduled and communicated earlier. Repeated home visits were made to deal with missed data using the permanent MHDSS site house number to identify missed households as well as data collectors training with practical exercises on how to avoid non-response were the major efforts to achieve complete participant enrolment.

### Data quality assurance

Prior to the actual data collection, emphasis was given in designing the study tool and training was given for data collectors with a practical exercise on how to collect the required data as well as the study tool was pretested in the nearby district, which was not part of the study area, and the necessary amendments were done on the flow and clarity of some questions to suit respondents. During the actual data collection, data collection manual (Provided in additional file 2) was used by each data collector to facilitate the data collection process and about 5% of the already surveyed households were randomly selected and re-interviewing took place at the time of data collection as cross-checking mechanism to ensure the validity of the collected data. In addition, all data were checked for completeness, accuracy, and consistency prior to data entry.

### Statistical methods

Since the problem of missing data was avoided by making special efforts a “complete-case” analysis approach was performed using the Statistical Package for Social Sciences. Backward stepwise logistic regression analysis technique was applied to identify factors linked with childhood ALRI by controlling potential confounders simultaneously. All statistical tests were two-sided with *p*-value < 0.05 considered statistically significant, and an adjusted odds ratio (AOR) with respective confidence interval was used as measures of effect.

Concerning model fitness, once the model has been fitted to the data, the Hosmer & Lemeshow test was checked and the model is a good fit for the data (*P = 0.347*) [31]. Besides Hosmer & Lemeshow test, the goodness-of-fit was also evaluated using a receiver operating characteristic (ROC) curve analysis and the model classifies the group significantly better than by chance with a ROC curve value of 0.735 (95% CI: 0.719–0.752). In addition, a multicollinearity diagnosis was carried out using the correlation matrix method, and all values were well below 0.20, which confirmed that multicollinearity among the predictor variables was not a concern for the model.

Furthermore, interaction effect test was performed between some of the independent factors, by adding a term for the product of the two factors to the regression model to assess the interaction effects on the outcome. As a final point, both data analysis and interpretations were made at the child level and the paper is reported following the STROBE (Strengthening the Reporting of Observational Studies in Epidemiology) statement to increase its quality and address the essential components of the report [32].

## Results

### Socio-demographic characteristics of study participants

A total of 5830 eligible children were included in the study within 100 clusters. Out of which 51.7% were male. Most mothers/caretakers were married (92.7%), do not have formal education (79.1%) and about two-thirds (66.8%) of them were farmers by occupation. About one-third of children (34.7%) live in households with four up to five family members as shown in Table 1.

**Table 1:** Socio-demographic characteristic of study households in Northwest Ethiopia, May 2018

Socio demographic characteristic	Frequency	Percent
Response rate	5830	100
Gender of child		
Female	2816	48.3
Male	3014	51.7
Age of child		
< 1 Year old (0–11 months)	1601	27.5
1 Year old (12–23 months)	1664	28.5
2 Years old (24–35 months)	1528	26.2
3 Years old (36–47 months)	1037	17.8
Age of mothers/ caretakers		
16–25 Years	1318	22.6
26–35 Years	2854	49.0
36–45 Years	1438	24.7
46–55 Years	180	3.1
> 56 Years	40	0.6
Marital status of mothers/ primary caretakers		
Married	5406	92.7
Single	120	2.1
Divorced	232	4.0
Separated	53	0.9
Widowed	19	0.3
Educational status of mothers/ caretakers		
Do not have formal education	4610	79.1
Primary school (grade 1–8)	474	8.1
Secondary school (grade 9–12)	366	6.3
Higher education	380	6.5
Religion of mothers/ caretakers		
Orthodox	5699	97.8
Muslim	123	2.1
Protestant	8	0.1
Occupational status of mothers/ caretakers		
Farmer	3893	66.8
Merchant	839	14.4
Housewife	496	8.5
Employee	332	5.7
Daily laborer	234	4
Student	36	0.6
Total family size of the household		
2–3	1158	19.9
4–5	2025	34.7
6–7	1784	30.6
8–9	746	12.8
10 or more	117	2.0

### Cooking related characteristics of study households

The majority of the households use the traditional type of “Injera” baking (94.5%) and cooking (92.1%) stoves. All study children live in households that rely principally on high pollution biomass fuels (wood/shrub/straw (80%), charcoal (14.4%) and cow dung (5.6%) for cooking as shown in Table 2. Besides, the presence of extra indoor (95.8%) and outdoor (38.1%) burning events such as coffee ceremony, burning incense, local alcohol/ “*areqi*” making, burning rubbish and charcoal production were common observable facts in the study area as revealed in Table 2.

**Table 2:** Main cooking quarter characteristics of study households in Northwest Ethiopia, May 2018

Socio demographic characteristic	Frequency	Percent
Location of main cooking quarter		
Inside the living house	2145	36.8
Separate kitchen	3685	63.2
“Injera” baking stove type		
Traditional biomass stove	5508	94.5
Improved biomass stove	322	5.5
Cookstove type		
Traditional stove	5371	92.1
Improved stove	459	7.9
Cooking fuel type		
Wood/shrub/straw	4662	80.0
Cow dung	328	5.6
Charcoal	840	14.4
Type of lamp for household lighting		
Electric/Solar	3760	64.5
“*Masho*”/Candle/ “*Fanos*”	1009	17.3
“*Kuraz*”	979	16.8
Wood	82	1.4
Extra indoor burning event		
Yes	5583	95.8
No	247	4.2
Type of extra indoor burning event		
Coffee ceremony & incense burning	4166	74.6
Local alcohol/“*areqi*” making	1417	25.4
Outdoor burning event		
Yes	2224	38.1
No	3606	61.9
Type of outdoor burning event		
Burning rubbish	881	15.1
Charcoal production	549	9.4
local alcohol/” *areqi*” making	601	10.3
Cooking for business	193	3.3
Secondary smoking		
Yes	104	1.8
No	5726	98.2

### Prevalence of childhood acute lower respiratory infection

The prevalence of childhood ALRI was 19.2% (95% CI: 18.2–20.2). ALRI distribution by gender was found to be 20% for females and 18.4% for male children. The distribution by age group was found to be 21.9% for those aged less than 1 year, 19.7% for 1 year, 18% for 2 years and 16% for 3 years old children as indicated in Table 3.

**Table 3:** Childhood ALRI distribution by gender and age in Northwest Ethiopia, May 2018

Gender of child	Age	ALRI status		Total
		No (%)	Yes (%)	
Female	< 1 Year old (0–11 months)	622 (78.2)	173 (21.8)	795
	1 Year old (12–23 months)	628 (78.9)	168 (21.1)	796
	2 Years old (24–35 months)	584 (80.9)	138 (19.1)	722
	3 Years old (36–47 months)	418 (83.1)	85 (16.9)	503
	Sub-total	2252 (80)	564 (20)	2816
Male	< 1 Year old (0–11 months)	629 (78)	177 (22)	806
	1 Year old (12–23 months)	708 (81.6)	160 (18.4)	868
	2 Years old (24–35 months)	669 (83)	137 (17)	806
	3 Years old (36–47 months)	453 (84.8)	81 (15.2)	534
	Sub-total	2459 (81.6)	555 (18.4)	3014
Both genders	< 1 Year old (0–11 months)	1251 (78.1)	350 (21.9)	1601
	1 Year old (12–23 months)	1336 (80.3)	328 (19.7)	1664
	2 Years old (24–35 months)	1253 (82)	275 (18)	1528
	3 Years old (36–47 months)	871 (84)	166 (16)	1037
	Total	4711 (80.8)	1119 (19.2)	5830

### Factors associated with childhood acute respiratory infection

The effect of several factors on childhood ALRI status was evaluated by fitting the logistic regression model and a narrative description of the major results is presented next. To start with the findings of socio-demographic factors, the female gender was found to significantly increase the odds of childhood ALRI than the male with an estimated AOR of 1.17 (95% CI: 1.02–1.35), and the odds of childhood ALRI was found to decrease by 41% among three-years-old children than under one-year-old children with an estimated AOR of 0.59 (95% CI: 0.48–0.74). However, children whose mothers’ age were between 26 and 35, 36–45, 46–55 and above 56 years old did not show a significant association with the odds of childhood ALRI with AOR of 1.30 ((95% CI: 1.08–1.57), 1.39 ((95% CI: 1.13–1.72), 1.95 ((95% CI: 1.32-, 2.89) and 2.41 (95% CI: 1.12–5.18) respectively when compared with children whose mothers’ age was between 16 and 25 years old as depicted in Table 4.

**Table 4:** Multivariable analysis of factors associated with childhood ALRI in Northwest Ethiopia, May 2018

Characteristics	ALRI status		Crude OR (95% CI)	AOR (95% CI)
	No	Yes		
Gender of child				
Female	2252	564	1.11 (0.97,1.26)	1.17 (1.02,1.35)
Male^a^	2459	555	1.0	1.0
Age of child				
< 1 Year old^a^	1251	350	1.0	1.0
1 Year old	1336	328	0.88 (0.74,1.04)	0.78 (0.65, 0.94)
2 Years old	1253	275	0.78 (0.66,0.94)	0.63 (0.52,0.77)
3 Years old	871	166	0.68 (0.56,0.84)	0.59 (0.48, 0.74)
Age of mother/ caregiver				
16–25 Years^a^	1106	212	1.0	1.0
26–35 Years	2304	550	1.25 (1.05,1.48)	1.30 (1.08,1.57)
36–45 Years	1141	297	1.36 (1.12,1.65)	1.39 (1.13, 1.72)
46–55 Years	131	49	1.95 (1.36,2.80)	1.95 (1.32,2.89)
> 56 Years	29	11	1.98 (0.97,4.02)	2.41 (1.12,5.18)
Child vaccination				
Yes	3605	742	0.60 (0.52,0.70)	0.60 (0.51, 0.70)
No^a^	1106	377	1.0	1.0
Breast-feeding				
Exclusive	3191	627	0.61 (0.53,0.69)	0.66 (0.57, 0.77)
No or partial^a^	1520	492	1.0	1.0
Family ALRI history				
Yes	988	317	1.49 (1.29,1.73)	1.36 (1.15, 1.59)
No^a^	3723	802	1.0	1.0
Number of rooms in living house				
1 room^a^	1050	334	1.0	1.0
2 rooms	2608	595	0.72 (0.62,0.84)	0.45 (0.37,0.54)
3 rooms	930	171	0.58 (0.47,0.71)	0.45 (0.35, 0.57)
4 or more rooms	123	19	0.49 (0.30,0.80)	0.38 (0.22,0.65)
Location of cooking quarter				
Inside living house	1632	513	1.60 (1.40,1.82)	1.36 (1.16,1.59)
Separate kitchen^a^	3079	606	1.0	1.0
Chimney				
Yes	2043	340	0.57 (0.50,0.66)	0.60 (0.51,0.70)
No^a^	2668	779	1.0	1.0
Eaves space				
Yes	2442	460	0.65 (0.57,0.74)	0.70 (0.60,0.84)
No^a^	2269	659	1.0	1.0
“Injera” baking stove type				
Traditional^a^	4425	1083	1.0	1.0
IBS	286	36	0.51 (0.36,0.73)	0.78 (0.52, 1.15)
Frequency of “Injera” baking event				
One or more per day	656	224	1.0	1.0
Every other day	501	120	0.70 (0.55,0.90)	0.74 (0.56,0.98)
Every 3 days	3428	753	0.64 (0.54,0.76)	0.66 (0.55,0.81)
Every 4 or more days	126	22	0.51 (0.32,0.82)	0.60 (0.36,1.00)
Cookstove type				
Traditional^a^	4304	1067	1.0	1.0
Improved	407	52	0.52 (0.38,0.69)	0.43 (0.28,0.67)
Cooking fuel type				
Cow dung	225	103	2.80 (2.07,3.80)	1.54 (1.02,2.33)
Wood/ straw/ shrub	3764	898	1.46 (1.19,1.80)	1.10 (0.79,1.53)
Charcoal^a^	722	118	1.0	1.0
Frequency of meal cooked per day				
One or less^a^	914	176	1.0	1.0
Two	2764	602	1.13 (0.94,1.36)	1.22 (0.99,1.51)
Three	758	250	1.71 (1.38,2.13)	1.46 (1.14,1.87)
Four/more	275	91	1.72 (1.29,2.29)	1.55 (1.13,2.13)
Cooking time per day				
1–2 h^a^	1299	218	1.0	1.0
3–4 h	2905	717	1.47 (1.25,1.74)	1.54 (1.28,1.86)
5 or more hours	507	184	2.16 (1.73,2.70)	1.75 (1.37,2.24)
Child near stove				
Yes	4200	1042	1.65 (1.28,2.11)	1.41 (1.06,1.88)
No^a^	511	77	1.0	1.0
Secondary smoking				
Yes	71	33	1.99 (1.31,3.02)	2.11 (1.32,3.36)
No^a^	4640	1086	1.0	1.0
Extra indoor burning event				
Yes	4492	1091	1.90 (1.28,2.83)	2.19 (1.41,3.40)
No^a^	219	28	1.0	1.0
Outdoor burning event				
Yes	1651	573	1.95 (1.71,2.22)	1.69 (1.06,2.70)
No^a^	3060	546	1.0	1.0

^a^reference category

With reference to household ventilation related factors, the odds of childhood ALRI was found to decrease among children living in a household with a chimney than children living without a chimney with an estimated AOR 0.60 (95% CI: 0.51–0.70). The odds of childhood ALRI were also found to decrease among children living in a household with an eaves space than children living without an eaves space with estimated AOR of 0.70 (95% CI: 0.60–0.84). Children living in homes with four/more rooms were found to decrease the odds of childhood ALRI with an estimated AOR of 0.38 (95% CI: 0.22–0.65) when compared with children living in one room homes as shown in Table 4.

Concerning household cooking technology-related factors, the odds of childhood ALRI was not showed a significant association with type of “Injera” baking stove, children living in homes with IBS when compared with children living in homes with TBS type with estimated AOR of 0.78 (95% CI: 0.52–1.15). It showed, however, a significant association with type of cookstove and the odds of childhood ALRI was found to decrease by 57% among children living in homes with an ICS when compared with children living in homes with traditional cookstove [AOR = 0.43 (95% CI: 0.28–0.67)]. Besides the cookstove technology type, the odds of childhood ALRI showed a significant association with the type of fuel. Children living in cow dung using households as primary cooking fuel type were found to increase the odds of childhood ALRI with an estimated AOR of 1.54 (95% CI:1.02–2.33) than children living in charcoal using homes as revealed in Table 4.

Concerning the findings of behavioral factors, children who were reported to spend some time near stove during cooking were found to increase the odds of childhood ALRI than children who do not spend time near the cookstove with AOR of 1.41 (95% CI: 1.06–1.88). Similarly, presence of extra indoor burning events such as coffee ceremony, burning incense and production of local alcohol/“*Areqi*” in the household was associated with more than two-fold increase in the odds of suffering from childhood ALRI with an estimated AOR of 2.19 (95% CI:1.41–3.40) when compared with no extra indoor burning event in the household. Besides the indoor burning event, presence of outdoor burning events such as charcoal and local alcohol/” *areqi*” production near the household was found to increase the odds of childhood ALRI with an estimated AOR of 1.69 (95% CI: 1.06–2.70) when compared with no outdoor burning event. Concerning frequency of meals cooked per day, children living in homes who commonly cooked three and four/more meals per day were found to increase the odds of childhood ALRI than children living in households who cooked one or less meal per day with AOR of 1.46 (95% CI: 1.14–1.87) and 1.55 (95% CI: 1.13–2.13) respectively. The odds of childhood ALRI was found to decrease among children who were exclusively breast-fed for the first 6 months of life than children who were not breast-fed with an estimated AOR of 0.66 (95% CI: 0.57–0.77). In the present study, the odds of suffering from childhood ALRI were also elevated by more than twice for secondary smoking with AOR of 2.11 (95% CI: 1.32–3.36) as presented in Table 4.

Finally, a variety of interaction terms were created and tested between some of the independent factors. Nevertheless, our models did not significantly capture any interaction effect between the cooking-related factors such as “Injera” baking stove type and indoor burning event as well as between cookstove type and average cooking time per day.

## Discussion

This study investigated the occurrence of community-acquired childhood ALRI and associated factors as part of the ongoing trial project on the impact of improved “Injera” baking stove use on childhood ALRI prevention in Northwest Ethiopia. The entire study households used high pollution biomass fuels as the primary household energy source in the form of wood/shrub/straw (80%), charcoal (14.4%) and cow dung (5.6%). This finding is similar with previous studies conducted in rural households of Ethiopia which reported 99.9% [28], 100% [10] and 95% [33] biomass fuel use. In addition, a recent study has also reported that only 12.6% of the households used clean fuel energy sources in Southern Ethiopia [34]. Correspondingly, a study in Ghana also indicated that 99% of the households use biomass as their primary fuel source [35] and according to the global solid-fuel use estimation, 2.8 billion people still burn solid fuels for cooking [36]. However, HAP exposure from cooking with biomass fuels has been linked with childhood pneumonia [37, 38] and an up to date estimate of the disease burden of HAP suggests that every year, HAP from biomass fuel use causes 455,000 ALRI deaths and an ALRI population attributable fraction of 52% globally [39]. The use of unclean fuel for household cooking was also found to elevate the odds of childhood ARI in South Ethiopia with an AOR of 2.09 (95% CI: 1.03–4.22) [34]. Furthermore, excess biomass fuel harvesting has been also linked with environmental implications through deforestation and soil productivity in North Ethiopia [40].

Therefore, given cooking with biomass fuels has been linked with childhood ALRI and environmental pollution, this complete reliance on biomass fuels might expose children to a high risk of ALRI in the study area, and the use of cleaner fuels could be the most important intervention to reduce ALRI among children, who are often most at risk for HAP exposure in particular [41]. The finding of this study has important implications for public health and environmental programs and can be used to formulate public health and environmental policies towards addressing childhood ALRI morbidity and mortality from HAP exposures as well as mitigating climate change.

Concerning the prevalence of childhood ALRI, the result of this study demonstrates a higher prevalence of childhood ALRI (19.2%; 95% CI: 18.2–20.2). This finding is somewhat in agreement with two previous studies which reported a prevalence of 21% [10] and 23.9% [12] in South and central Ethiopia respectively. However, the finding of this study is found to be contradictory with the finding of other previous cross-sectional studies. On one hand, it is lower than the finding of two studies which reported a prevalence of 33.5% [8] and 26.3% [9] in South and Northwest Ethiopia respectively; on the other hand, it is higher than the finding of a study which reported a prevalence of 16.1% [11] in Northwest Ethiopia. This disagreement might be partly explained by a difference in socio-demographic characteristics, climatic zone, and local cooking characteristics as well as seasonal variation of the studies undertaken. The availability of this finding could drive commitments by stakeholders to identify ALRI as a priority health problem and seek appropriate solutions to reduce its burden particularly among children in Ethiopia and other LMICs.

Regarding the factors associated with childhood ALRI, discussions of the major findings are presented next in four categories as socio-demographic, household ventilation, cooking technology, and behavioral factors. To start with the socio-demographic factors, the female gender was found to significantly increase the odds of childhood ALRI than the male gender with an estimated AOR of 1.17 (95% CI: 1.02–1.35). Similarly, a previous study conducted in Brazil among under 5 years old children reported that male gender constituted a protective factor against child pneumonia with an estimated AOR of 0.53 [42]. The function of sex in the incidence of childhood community-acquired pneumonia remains unclear [43, 44] and more research is needed for a better explanation of this gender disparity in the risk of childhood ALRI.

Concerning household ventilation related variables; the finding suggests that children living in a household with a chimney were found to decrease the odds of childhood ALRI by 40% than children living without a chimney with estimated AOR of 0.60 (95% CI: 0.51–0.70). The reason for this result could be a difference in HAP exposure caused by difference in stove venting using a chimney for removing indoor contaminants and it was fairly in concurrence with a study conducted in Southern part of Ethiopia which report the absence of chimney as a risk factor for childhood ARI (*P* < 0.05) [10]. Similarly, previous randomized controlled trial also reported a 22% reduction in childhood pneumonia from chimney stove intervention in Guatemala [26]. Hence, this considerable link between the use of chimney and childhood pneumonia risk reduction suggests the need for chimney intervention to reduce childhood pneumonia in the study area.

Concerning household cooking technologies, the odds of childhood ALRI did not show a significant association among children living in households with IBS when compared with children living in households with TBS type with an estimated AOR of 0.78 (95% CI: 0.52–1.15). The possible rationalization for this finding might be the currently distributed biomass-fuelled IBS technologies in Ethiopia may not be improved enough to reduce the odds of childhood ALRI when compared with the biomass-fuelled TBS type. The relationship needs to be further investigated using a more rigorous design with direct measures of HAP exposure and childhood ALRI outcome. Such research is important because large-scale initiatives for the dissemination of IBSs are still underway in Ethiopia [45, 46], and perhaps, no community-level biomass IBS trial research had been conducted [16–18] and health effect of the fuel-efficient IBS [46] is unknown.

Concerning cookstove technology, the odds of childhood ALRI was found to decrease by 57% among children living in homes with an ICS technology when compared with children living in homes with traditional cookstove [AOR = 0.43 (95% CI: 0.28–0.67)]. This finding was somewhat in line with a study conducted in slum households of Addis Ababa in Ethiopia, which showed children living in traditional stove using homes were found 6 times more likely to suffer from ALRI than that of the improved stove using homes with AOR of 6.07 (95% CI: 1.62–22.76) [12]. Likewise, a recent stove trial reported that an improved cookstove (ICS) intervention was not able to reduce the risk of pneumonia among young children in rural Malawi with an incidence rate ratio of 1·01 [22]. In contrast to our finding, a randomized controlled trial reported a significant association of childhood pneumonia reduction (RR 0·82; 0·70, 0·98) among the intervention group [26]. This difference in the effectiveness of ICSs might be explained by a difference in the ethnic group, geographical location, local fuel resources and seasonal variations of the studies undertaken [47].

The other finding worth highlighting is related to fuel type, the odds of childhood ALRI was also found significantly linked with primary household cooking fuel type. Children living in cow dung fuel using homes were found to increase the odds of childhood ALRI than children living in charcoal using homes [AOR = 1.54 (95% CI: 1.02–2.33)]. The potential explanation for this finding might be different household fuel types result in different toxicities, with HAP from animal dung being extremely toxic [48, 49]. Hence, the use of cleaner stoves and fuels would be the most important cooking technology related intervention to reduce ALRI among children, and this finding can have important implications for public health programs and provides the evidence needed by policy-makers regarding the benefits of ICS and clean fuel technology adoptions on prevention of childhood ALRI.

Pertaining to the findings of behavioral factors, children who were reported to spend some time near cookstove during cooking were found to raise the odds of childhood ALRI than children who did not spend time near the cookstove with AOR of 1.41 (95% CI: 1.06–1.88). The possible explanation for the difference in childhood ALRI risk might be the reflection of the difference in HAP exposure caused by differences in distance from source and length of exposure time. Likewise, a significant association of childhood ALRI was reported with the carrying of a child while cooking with AOR of 1.66 (95% CI: 1.18–2.34) in South Ethiopia [34]. This finding implies that the practice of keeping children near cooking areas, carried on the back or placed near their mother to sleep, is a common practice in Ethiopia, and they became more at risk of ALRI associated with more proximal exposure of HAP [12, 34]. Hence, modifying child handling practices might be effective to decrease the risk of childhood ALRI from proximal exposure of HAP.

The other noteworthy behavioral finding was the effect of extra indoor burning events. Presence of extra indoor burning events, such as coffee ceremony, burning incense and local alcohol/” *areqi*” making in the household, was associated with more than two-fold increase in the odds of suffering from childhood ALRI with an estimated AOR of 2.19 (95% CI:1.41–3.40) when compared with no extra indoor burning event. This might be due to an increase in indoor particulate matter concentration as stated by a pilot study conducted among coffee ceremony participants in Addis Ababa homes which reported that the Ethiopian coffee ceremony inside houses could increase the concentration of HAP [50]. The most important inference of this finding is that HAP exposure from extra indoor burning events such as coffee ceremony, burning incense and local alcohol/“*areqi*” making in the household might elevate the odds of suffering from ALRI in children and this should be further investigated to estimate the separate childhood risks and recommend possible interventions.

The other finding worth highlighting is related to the frequency of meals cooked per day, children living in households who commonly cooked three and four/more meals per day were found to increase the odds of childhood ALRI with AOR of 1.46 (95% CI: 1.14–1.87) and 1.55 (95% CI: 1.13–2.13) respectively than children living in households who cooked one or less meal per day. This might be due to high HAP from frequent cooking events which are thought to be a predictor of HAP as reported by a large sample size longitudinal study conducted in rural Ethiopia [51]. In the typical context, this frequent cooking practice might determine both HAP exposure and resulting ALRI risk among under 4 years old children due to several periods of intense cooking smoke exposure per day as evidenced by previous studies conducted in Ethiopia [12, 34]. Hence, modifying child handling practices might be effective to decrease the risk of childhood ALRI from frequent exposure of HAP per day.

In the present study, breastfeeding for the first 6 months was found to decrease the odds of childhood ALRI by about 34% with an estimated AOR of 0.66 (95% CI: 0.57–0.77). This finding was in agreement with a case-control study in Southwest Ethiopia which reported that non-exclusive breastfeeding during the first 6 months of life increases the odds of childhood pneumonia by more than three-fold [29]. This might be explained by the protective effect of exclusive breastfeeding on incidence of childhood pneumonia as evidenced by previous studies [30, 52].

The odds of suffering from childhood ALRI was elevated by more than twice for secondary smoking with AOR of 2.11 (95% CI: 1.32–3.36) in the present study. In the same line, a facility-based study carried out in Southwest Ethiopia reported nearly a two-fold increase in community-acquired pneumonia among 2–59 months old children associated with current parental cigarette smoking with OR of 1.9 (95% CI: 1.1–3.7) [29]. In addition, a previous study also showed that second-hand smoke exposure, particularly from household tobacco smoke, increases the risk of lower respiratory tract infections in children [53], and that indoor smoking cessation might be effective to decrease the risk of childhood ALRI.

Concerning interaction effects between cooking-related factors, a variety of interaction terms were created and tested between some of the independent factors, by adding a term for the product of the two factors to the regression model. Nevertheless, our models did not significantly capture any interaction effect between the cooking-related factors such as “Injera” baking stove type and indoor burning event as well as between cookstove type and average cooking time per day. This finding might be explained by the fact that interaction effect might not always be noted, or it might not be captured adequately by our models due to the complex nature of the interaction effects s between factors [54], and this should be investigated further in future childhood ALRI risk studies for a better estimation of the relationship between household cooking-related factors and childhood ALRI outcome.

### Generalisability

Concerning the generalisability (external validity) of results, this study aimed to estimate the prevalence and factors associated with community-acquired childhood ALRI using the standard definition of Integrated Management of Childhood Illnesses (IMCI) algorithm. Besides, our study sample size was large and similar to the wider population of biomass fuel using households in the study area, which is representative of the biomass fuel user communities in Ethiopia. Moreover, the pragmatic approach that we used could ensure the generalisability of the study findings to the wider population by maintaining the external validity of results. Hence, the findings of this study could be generalized to biomass fuel user communities in Ethiopia and other LMICs.

### Strength and limitation

Our study has several strengths such as; it was community-based with a large sample size, application of data collection manual and cross-checking mechanisms were among others. This study is also subject to the limitations of survey questionnaires, including recall bias, and the point prevalence estimate which might be influenced by the season during which the survey took place. Besides, although the definition used for this study is intended to quantify childhood ALRI using the standard childhood pneumonia algorism [19, 20], childhood acute upper respiratory illness may have been included in the reported prevalence.

## Conclusion

High prevalence of childhood ALRI was demonstrated by this study which was associated with factors such as household ventilation (chimney & eaves space), cooking technologies (stove and fuel type) and household behavior (child handling and extra indoor cooking events). The findings of this large-sample study are generally consistent with the results obtained from previous studies. Thus, stakeholders shall work further to reduce childhood ALRI from HAP exposure through enhancing ventilation, making the transition to clean cooking technology as well as through behavioral changes in child handling and cooking patterns. In addition, considering interaction effects in future childhood ALRI risk studies is recommended for a better understanding and estimation of the relationship between household cooking-related factors and childhood ALRI. Conducting further study is also imperative to better understand how HAP reduction interventions are effective to reduce the health risks among the most susceptible segment of the population such as children.

## Supplementary information

**Additional file 1.** Data collection questionnaire (English-version).

**Additional file 2.** Data collectors’ manual (English-version).

## Data Availability

The data that support the findings of this study are available from the corresponding author on reasonable request and with permission of the MHDSS research center at Bahir Dar University in Ethiopia.

## Abbreviations

ALRI: Acute lower respiratory infection
AOR: Adjusted odds ratio
EDHS: Ethiopian Demographic and Health Survey
HAP: Household air pollution
IBS: Improved baking stove
ICS: Improved cookstove
IMCI: Integrated Management of Childhood Illnesses
LMICs: Low-and middle-income countries
MHDSS: Mecha Health and Demographic Surveillance System
SPSS: Statistical Package for Social Science
TBS: Traditional baking stove
TCS: Traditional cookstove
WHO: World Health Organization
