# Supplementary material for: Prevalence and risk factors of acute lower respiratory infection among children living in biomass fuel using households: a community-based cross-sectional study in Northwest Ethiopia
Source: BMC Public Health. 2020 Mar 19;20:363. doi: 10.1186/s12889-020-08515-w (PMC7083007; doi:10.1186/s12889-020-08515-w)
Supplement: Supplementary file 1 — Additional file 1. Data collection questionnaire (English-version). [file 12889_2020_8515_MOESM1_ESM.docx]

Additional file 1: Data collection questionnaire (English-version)

BAHIR DAR UNIVERSITY, COLLEGE OF MEDICINE AND HEALTH SCIENCES

SCHOOL OF PUBLIC HEALTH

Data collection questionnaire to assess prevalence and risk factors of acute lower respiratory infection among children living in households of “Mecha” Health and Demographic Surveillance System Sites, Northwest Ethiopia.

1. Introduction and parental written informed consent process instruction

Before beginning the data collection, introduce yourself and explain the objectives of the survey and how long the process might take. Remember, participants have the right to refuse to be interviewed. Next, with good communication identify the mother or the primary caretaker of the index child; this person will be the respondent. Please remember to explain the purpose, procedures, risks, benefits, and confidentiality as well as to complete a written informed consent of the interviewee (signature/thumb-print) before beginning the data collection. Then, circle all the possible answers of the respondent from the choice provided or put the answer of the respondents in the provided blank space without giving any option and restriction to the respondents using the following questionnaire.

| 1. Fill the following household location-related questions | | |  |
| --- | --- | --- | --- |
| *No* | *Questions* | *Response* |  |
|  | What is the date of the visit? | dd/mm/yy:____/____/______________ |  |
|  | What is the name of the sub-district (“Kebele”)? | ______________________________ |  |
|  | What is the name of the cluster/*“Got”*? | ______________________________ |  |
|  | What is the MHDSS household ID number? | \| Number \|  \| \| --- \| --- \| |  |
| 1. Fill the following socio-demographic characteristics | | | |
| *No* | *Questions* | *Response* | |
|  | What is the relationship of the primary caretaker with the index child? | \| Mother \| 1 \| \| --- \| --- \| \| Caretaker \| 2 \| | |
|  | What is the child’s gender? | \| Female \| 1 \| \| --- \| --- \| \| Male \| 2 \| | |
|  | What is the index child age? | \| <1 Year old (0-11 months) \| 1 \| \| --- \| --- \| \| 1 Year old (12-23 months) \| 2 \| \| 2 Years old (24-35 months) \| 3 \| \| 3 Years old (36-47 months) \| 4 \| | |
|  | What is the age of the index child’s mother or primary caretaker? | \| <15 Years \| 1 \| \| --- \| --- \| \| 16-25 Years \| 2 \| \| 26-35 Years \| 3 \| \| 36-45 Years \| 4 \| \| 46-55 Years \| 5 \| \| > 56 Years \| 6 \| | |
|  | What is the marital status of the index child’s mother or primary caretaker? | \| Married \| 1 \| \| --- \| --- \| \| Single \| 2 \| \| Divorced \| 3 \| \| Separated \| 4 \| \| Widowed \| 5 \| | |
|  | What is the educational status of the index child’s mother or primary caretaker? | \| Do not have formal education \| 1 \| \| --- \| --- \| \| Primary school (grade 1-8) \| 2 \| \| Secondary school (grade 9-12) \| 3 \| \| Higher education \| 4 \| | |
|  | What is the religion of the index child’s mother or primary caretaker? | \| Orthodox \| 1 \| \| --- \| --- \| \| Muslim \| 2 \| \| Protestant \| 3 \| \| Other \| 4 \| | |
|  | What is the occupational status of the index child’s mother or primary caretaker? | \| Farmer \| 1 \| \| --- \| --- \| \| Merchant \| 2 \| \| Housewife \| 3 \| \| Employee \| 4 \| \| Daily laborer \| 5 \| \| Student \| 6 \| \| Other \|  \| | |
|  | What is the total family size of the index child’s family? | \| Number \|  \| \| --- \| --- \| | |

| 1. Fill the following health-related characteristics of the index child | | | | |
| --- | --- | --- | --- | --- |
| N^o^ | | Questions | | Response |
|  | | Did the index child completely vaccinated for his/her age according to the guideline? *(Check for each type of immunization status on the immunization card, If a card is not available, use probing questions to find out if the child received that vaccination).* | | \| Yes \| 1 \| \| --- \| --- \| \| No \| 2 \| \| Do not know \| 99 \| |
|  | | What was/is the breastfeeding practice of the mother or primary caretaker during the first 6 months of life of the index child? | | \| Exclusive breastfeeding \| 1 \| \| --- \| --- \| \| No or partial breastfeeding \| 2 \| \| Do not know \| 99 \| |
|  | | Did the index child had/have an illness related to the symptoms of acute lower respiratory infection (ALRI) currently or at any time in the last two weeks? | | \| Yes \| 1 \| \| --- \| --- \| \| No \| 2 \| |
|  | | If the answer to question 17 is “Yes”, is the child’s respiratory illness pneumonia? (Further further ask the respondents to identify childhood pneumonia. In addition, if the child is still ill, assess the sick child using the Integrated Management of Neonatal and Childhood Illness algorism to identify childhood pneumonia. | | \| Yes \| 1 \| \| --- \| --- \| \| No \| 2 \| |
|  | | Did any member of the index child’s family have an illness related to the symptoms of ALRI at any time in the last two weeks? | | \| Yes \| 1 \| \| --- \| --- \| \| No \| 2 \| |
| 1. Fill the following main living house, and cooking quarter characteristics of the household | | | | |
| N^o^ | Questions | | Response code | |
|  | What is the number of rooms in the main living house? | | \| 1 room* \| 1 \| \| --- \| --- \| \| 2 rooms \| 2 \| \| 3 rooms \| 3 \| \| 4 or more rooms \| 4 \| | |
|  | Does the household have a main cooking quarter/ kitchen with a structure to be considered enclosed? | | \| Yes \| 1 \| \| --- \| --- \| \| No* \| 2 \| | |
|  | Where is the location of the main cooking quarter of the household? | | \| Inside the main living house \| 1 \| \| --- \| --- \| \| Separate kitchen \| 2 \| | |
|  | Does the main living house have hood/ chimney? (Observe) | | \| Yes \| 1 \| \| --- \| --- \| \| No* \| 2 \| | |
|  | What is the wall material of the main cooking quarter? (observe) | | \| Block/brick/ stone \| 1 \| \| --- \| --- \| \| Mud \| 2 \| \| Wood \| 3 \| \| Tin \| 4 \| \| Other; specify_ \| 5 \| | |
|  | What is the floor material of the main cooking quarter? (observe) | | \| Earthen \| 1 \| \| --- \| --- \| \| Ceramic \| 2 \| \| Wood \| 3 \| \| Carpet \| 4 \| \| Cement \| 5 \| \| Other; specify_ \| 6 \| | |
|  | What is the type of roof material of the main cooking quarter? (observe) | | \| Thatch/grass \| 1 \| \| --- \| --- \| \| Corrugated iron sheet \| 2 \| \| Concrete/brick \| 3 \| \| Other; specify___ \| 4 \| | |
|  | Is there an open eaves space between the wall and the roof of the main baking/cooking quarter? (observe) | | \| Yes \| 1 \| \| --- \| --- \| \| No \| 2 \| | |
|  | Does the main cooking quarter have a roof leak? | | \| Yes \| 1 \| No \| 2 \| \| --- \| --- \| --- \| --- \| | |
|  | How many windows are in the main cooking quarter? (observe) | | \| Number \|  \| \| --- \| --- \| | |
|  | Are the windows in the main cooking quarter commonly opened? | | \| Not open at all (0-25%) \| 1 \| \| --- \| --- \| \| Half of the time (25-75%) \| 2 \| \| Open almost all of the time (75-100%) \| 3 \| \| Not applicable \| 98 \| | |

| Fill the following main living house, and cooking quarter characteristics … continued | | | |
| --- | --- | --- | --- |
| N^o^ | Questions | Response code | |
|  | What is the primary type of stove used for “Injera” baking purposes? *(Observe & circle only one stove type).* | \| Traditional biomass stove \| 1 \| \| --- \| --- \| \| Improved biomass stove \| 2 \| \| Electric stove \| 3 \| \| Other, specify ----------------- \| 4 \| \| Not applicable \| 98 \| | |
|  | What is the primary type of stove used for cooking purposes? *(observe & circle only one stove type)* | \| Traditional biomass stove \| 1 \| \| --- \| --- \| \| Improved biomass stove \| 2 \| \| Gas stove \| 3 \| \| Electric stove \| 4 \| \| Other, specify______ \| 5 \| | |
|  | What is the primary type of fuel used for cooking? *(circle only one fuel type)* | \| Wood/shrub/straw \| \| \| 1 \| \| --- \| --- \| --- \| --- \| \| Cow dung \| 2 \| LPG, biogas \| 5 \| \| Charcoal \| 3 \| Electricity \| 6 \| \| Kerosene \| 4 \| Natural gas \| 7 \| \| Other ,specify; ---------------------- \| \| \| 8 \| | |
| 1. Fill the following cooking pattern related characteristics of the household | | | |
|  | What is the common number of “Injera” baking events per day? | \| Once per day \| 1 \| \| --- \| --- \| \| Every other day \| 2 \| \| Every 3 days \| 3 \| \| Every 4 or more days \| 4 \| \| Not applicable \| 98 \| | |
|  | What is the common number of meals cooked per day? | \| 1 meal per day \| 1 \| \| --- \| --- \| \| 2 meals per day \| 2 \| \| 3 meals per day \| 3 \| \| 4 more meals/ day \| 4 \| \| Not applicable \| 98 \| | |
|  | What is the average cooking time in hours per day in the household? | \| 1-2 hours* \| 1 \| \| --- \| --- \| \| 3-4 hours \| 2 \| \| 5 or more hours \| 3 \| \| Not applicable \| 98 \| | |
|  | Does the index child regularly spend some moment in time near (within 1.5-meter distance) the cookstove during cooking? | \| Yes \| 1 \| \| --- \| --- \| \| No \| 2 \| | |
| 1. Fill the following alternative sources of household air pollution-related characteristics of the household | | | |
| N^o^ | Questions | | Response code |
|  | What is the type of lamp commonly used for household lighting purposes at night? | | \| Electric/Solar \| 1 \| \| --- \| --- \| \| “*Masho*”/Candle/“ *Fanos*” \| 2 \| \| “ *Kuraz*” \| 3 \| \| Wood \| 4 \| \| Other \| 5 \| |
|  | Does cigarette commonly smoked inside the main living house? | | \| Yes \| 1 \| \| --- \| --- \| \| No \| 2 \| |
|  | Is there an extra indoor burning event that commonly occurs inside the main living house or cooking quarter that may influence the indoor air quality as an alternative source of household air pollution? | | \| Yes \| 1 \| \| --- \| --- \| \| No \| 2 \| |
|  | If yes to the above question, what is the type of extra indoor burning event?  (circle all possible responses) | | \| Coffee ceremony with burning incense \| 1 \| \| --- \| --- \| \| Local alcohol/”*areqi*” making \| 2 \| \| Other ,specify:_____ \|  \| |
|  | Is there an extra outdoor burning event that commonly occurs nearby the main living house or cooking quarter that may influence the indoor air quality as an alternative source of household air pollution? | | \| Yes \| 1 \| \| --- \| --- \| \| No \| 2 \| |
|  | If yes to the above question, what is the type of extra outdoor burning event?  (circle all possible responses) | | \| Burning rubbish \| 1 \| \| --- \| --- \| \| Charcoal production \| 2 \| \| local alcohol/ ”*areqi*” making \| 3 \| \| Cooking for business \| 4 \| \| Other \| 5 \| |

Thank the interviewee for the information, and end the interview.

Interviewer name: _____________________; Sig: _________; Date of the interview: ________

Supervisor name: _______________________; Sig: _________ Date checked: ______________
