# Supplementary material for: Prevalence and risk factors of acute lower respiratory infection among children living in biomass fuel using households: a community-based cross-sectional study in Northwest Ethiopia
Source: BMC Public Health. 2020 Mar 19;20:363. doi: 10.1186/s12889-020-08515-w (PMC7083007; doi:10.1186/s12889-020-08515-w)
Supplement: Supplementary file 2 — Additional file 2. Data collectors’ manual (English-version). [file 12889_2020_8515_MOESM2_ESM.docx]

# Additional file 2: Data collectors’ manual (English-version)

BAHIR DAR UNIVERSITY, COLLEGE OF MEDICINE AND HEALTH SCIENCES

SCHOOL OF PUBLIC HEALTH

Data collectors’ manual/guide to assess prevalence and risk factors of acute lower respiratory infection among children living in households of “Mecha” Health and Demographic Surveillance System Sites, Northwest Ethiopia.

# Introduction

A community-based cross-sectional study will be employed for one year to assess the prevalence and risk factors of acute lower respiratory infection (ALRI) among children living in households of the “Mecha” Health and Demographic Surveillance System site. To estimate the prevalence of childhood ALRI and associated factors, data collection will be carried out through face-to-face interviews with a mother or primary caretaker of the index child using a structured questionnaire with several closed and open-ended questions as well as through direct verifications whenever essential and possible. The data collection tool/questionnaire contains household location, socio-demographic, index child health, main living house and cooking quarter characteristics, cooking pattern and alternative sources of household air pollution related questions.

# Study objective

The general objective of this study is to assess the magnitude and factors associated with childhood acute lower respiratory infection (ALRI) in the “Mecha” Health and Demographic Surveillance System site, Northwest Ethiopia.

# Purpose of the data collectors’ manual

This manual is designed to serve as a training and data collection reference tool for data collectors and supervisors participating in this study in the “Mecha” Health and Demographic Surveillance Site, Northwest Ethiopia. The manual is intended to explain the questions in the survey that require further clarification and provide the required knowledge to data collectors to perform their roles. However, for childhood illness of childhood acute lower respiratory infection (ALRI) screening; this manual should be used in conjunction with the Integrated Management of Neonatal and Childhood Illness (IMNCI) training manual.

# General information

All questions should be administered to a mother or primary caretaker of the selected index child, and the questions are arranged according to the sections under which they appear in the actual survey tool and the question numbers in this manual match those in the survey tool. The questions in the survey are a mix of open-ended questions and those where responses can be selected from a list provided. Thus, please pay particular attention to the instructions accompanying each question. Interviewers should NOT read response options to respondents after administering a question but should choose the response which best fits the information given by the respondent. If the information given is not enough to select a fitting response, the interviewer should neutrally ask for more information by asking further questions such as “could you please give me a bit more information?” etc.

For all questions, use the following codes to record missing data፡

| Response | Code |
| --- | --- |
| Not willing to tell | 0 |
| Not applicable/relevant | 98 |
| Do not know | 99 |

The data collection tool (questionnaire) is organized into seven major sections, in the following order:

- SECTION A: Introduction and parental written informed consent process instructions
- SECTION B: Household location related questions
- SECTION C: Socio-demographic characteristics
- SECTION D: Health-related characteristics of the index child
- SECTION E: Main living house, and cooking quarter related characteristics
- SECTION F: Cooking pattern related characteristics of the household
- SECTION G: Alternative sources of household air pollution related characteristics

# Data collection tool/questionnaire descriptions by section

## SECTION A: Introduction and parental written informed consent process

Before beginning the data collection, introduce yourself and explain the objectives of the survey and how long the process might take. Remember, that households have the right to refuse to be interviewed. Next, with good communication identify the mother or the primary caretaker of the index child; this person will be the respondent. Please remember to explain the purpose, procedures, risks, benefits, and confidentiality as well as to complete a written informed consent of the interviewee (signature/thumb-print) (See annex: 8) before beginning the data collection.

| SECTION B: Household location-related questions | | | |
| --- | --- | --- | --- |
| S/N^o^ | Questions | Descriptions | |
|  | What is the date of the visit? | Record the date of the visit using the format date/ month/year (dd/mm/yy) in the Gregorian calendar. | |
|  | What is the name of the sub-district (“Kebele”)? | Record the name of the sub-district (“Kebele”) | |
|  | What is the name of the cluster/*“Got”*? | Record the name of the cluster/*“Got”* | |
|  | What is the MHDSS household ID number? | Record the correct household ID number of the respondent’s given by Bahir Dar University under MHDSS. Please note that the household ID number should be recorded at the beginning and at the footer of every page to ensure that the information can be correlated to the relevant respondents during data cleaning and analysis. | |
| SECTION C: Socio-demographic characteristics | | | |
| S/N^o^ | Questions | Description | |
|  | What is the relationship of the primary caretaker with the index child? | Ask the relationship of the respondent with the index child and record as mother or caretaker. | |
|  | What is the child’s gender? | Ask the respondent and record the index child’s gender | |
|  | What is the index child age? | Ask the respondent and record the index child’s age. | |
|  | What is the age of the index child’s mother or primary caretaker? | Ask the respondent and record the current age of mothers/ caretakers | |
|  | What is the marital status of the index child’s mother or primary caretaker? | Ask and record the current marital status of the index child’s mother or primary caregiver | |
|  | What is the educational status of the index child’s mother or primary caretaker? | Ask and record the current educational status of the index child’s mother or primary caregiver | |
|  | What is the religion of the index child’s mother or primary caretaker? | Ask and record the religion of the index child’s mother or primary caregiver | |
|  | What is the occupational status of the index child’s mother or primary caretaker? | Ask and record the occupational of the index child’s mother or primary caregiver | |
|  | What is the total family size of the index child’s family? | Ask the respondent and record how many people live in the household. Remember, family means living independently, sharing the same roof & food. | |
| SECTION D: Health-related characteristics of the index child | | | |
| S/N^o^ | Questions | Description | |
|  | Did the index child completely vaccinated for his/her age? | Ask the respondent and record whether the index child has completely vaccinated (Diphtheria, tetanus, Pertussis, HB & H & influenza B, Pneumococcal conjugate, Rota, Polio & Measles) for his/her age according to the guideline. See the card or other document where vaccination status is written down and check each type of immunization status on the immunization card, If a card is not available, use probing questions to find out if the child received that vaccination. | |
|  | What was/is the breastfeeding practice of the mother or primary caretaker during the first 6 months of life of the index child? | Ask the respondent and record whether the index child exclusively breastfeed or not for the first 6 months of life. | |
|  | Did the index child had/have an illness related to the symptoms of acute lower respiratory infection (ALRI) currently or at any time in the last two weeks? | With good communication ask the index child’s mother, whether the child has an illness related to the symptoms of ALRI at any time in the last two weeks. Mothers may use such terms as “noisy”, “fast” or interrupted breathing to describe difficult breathing. | |
|  | If the answer to question 17 is “Yes”, was/is the child’s illness pneumonia? | If the index child has ALRI, further ask the respondents to identify childhood pneumonia. Besides, if the child is still ill, assess the sick child using the Integrated Management of Neonatal and Childhood Illness algorism to identify childhood pneumonia. | |
|  | Did any member of the index child’s family have an illness related to the symptoms of ALRI at any time in the last two weeks? | Ask the respondent and record whether any member of the family had an illness related to the symptoms of acute lower respiratory infection at any time in the last two weeks or not. | |
| SECTION E: Main living house, and cooking quarter related characteristics | | | |
| S/N^o^ | Questions | | Description |
|  | What is the number of rooms in the main living house? | | Count and record ONLY the number of living rooms in the main living house. |
|  | Does the household have a main cooking quarter/kitchen with a structure to be considered enclosed? | | First, select the area where the main cooking activity takes place, then observe and record the presence of a main cooking quarter/kitchen with a structure to be considered enclosed. |
|  | Where is the location of the main cooking quarter of the household? | | Observe and record the location of the main cooking quarter of the household. |
|  | Does the main cooking quarter (kitchen) have hood or chimney? | | Observe and record the existence of hood/chimney in the main cooking quarter (kitchen) |
|  | What is the wall material of the main cooking quarter? | | Observe and record the type of material from which the wall is made of. |
|  | What is the floor material of the main cooking quarter? | | Observe and record the type of materials from which the floor made of. It is important to be as accurate as possible as some materials will allow smoke to pass through |
|  | What is the type of roof material of the main cooking quarter? | | Observe and record the type of materials from which the roof is made of. |
|  | Is there an open eaves space between the wall and the roof of the main cooking quarter? | | Observe and record the presence of an open eaves space (more than 1 foot) between the wall and the roof of the main cooking quarter. |
|  | Does the main cooking quarter have a roof leak? | | Record the existence of a roof leak in the main cooking quarter by asking the respondent and observing. |
|  | How many windows are in the main cooking quarter? | | Observe and record the number of windows in the main cooking quarter if any. |
|  | Are the windows in the main cooking quarter commonly opened? | | Ask the respondent and record whether the windows in the main cooking quarter are commonly opened |
|  | What is the primary type of stove used for “Injera” baking purposes? | | Ask the respondent & determine only one primary stove type used for “Injera” baking among the list. If not possible to determine by the respondent, ask permission and observe the stove to determine the type. |
|  | What is the primary type of stove used for other cooking purposes? | | Ask the respondent and circle only one primary stove type used for other cooking/boiling water purposes. If not possible to determine by the respondent ask permission and observe the stove to determine the type. |
|  | What is the primary type of fuel used for other cooking/boiling water purposes? | | Ask the respondent and circle only one primary fuel type used for other cooking/boiling water purposes. |
| SECTION F: Cooking pattern related characteristics of the household | | | |
| S/N^o^ | Questions | Description | |
|  | What is the common number of “Injera” baking events per day? | Ask the respondent and circle/record the number of “Injera” baking events per day. | |
|  | What is the common number of meals cooked per day? | Ask the respondent and circle/record the number of meals cooked per day. | |
|  | What is the average cooking time in hours per day? | This question is aimed at getting information on the amount of time families spend in cooking. Thus, ask the respondent and record the time taken for cooking in hours per day on average. | |
|  | Does the index child regularly spend some moment in time near (within 1.5-meter distance) the cookstove during cooking times? | Ask the respondent and record whether the index child usually spends some moment in time within a 1.5-meter distance from the cookstove during backing/cooking times. | |
| SECTION G: Alternative sources of household air pollution-related characteristics | | | |
|  | What is the type of lamp commonly used for household lighting purposes at night? | Ask the respondent/observe and record the type of lamp commonly used for household lighting purposes at night. | |
|  | Does cigarette commonly smoked inside the main living or cooking quarter? | Ask the respondent and record whether any member of the family commonly smoke cigarette inside the main living or cooking quarter. | |
|  | Is there an extra indoor burning event that commonly occurs inside the main living house or cooking quarter that may influence the indoor air quality? | Ask the respondent and observe; and record whether an extra burning event occurs inside the main living house or cooking quarter that may influence the indoor air quality as alternative sources of household air pollution. | |
|  | If the answer to the above question is “Yes”, what is the type of extra indoor burning event? | If there is an extra indoor burning event, ask the respondent/ observe and record the type of extra indoor burning events such as the burning of incense, coffee ceremony, local alcohol making (“Areqi”) and cooking for business or other. | |
|  | Is there an extra outdoor burning event that commonly occurs nearby the main living/cooking quarter that may influence the indoor air quality? | Ask the respondent and observe, and record whether an extra outdoor burning event occurs nearby the main living/ cooking quarter that may influence the indoor air quality as alternative sources of household air pollution. | |
|  | If the answer to the above question is “Yes”, what is the type of extra outdoor burning event? | If there is an extra outdoor burning event, ask the respondent and observe; and record the type of extra outdoor burning events such as burning rubbish, charcoal production, cooking for business or other. | |

# Keeping the relevant records to be used in the study

For the purpose of this study, data collectors shall keep and maintain only one important form, that is, the questionnaire; and data shall be completed for every index child <4 years in all households under the selected clusters/*“Gots”* of “Mecha” Health and Demographic Surveillance System site.

# Reminder notes

- - The data collector must check whether the information on the form is consistent with the information required.
  - The data collector shall understand that supervisors and investigators will also check whether the information on the form is valid and consistent with the required information using all possible methods.
  - Give health information on prevention of acute lower respiratory infections for parents of every study child who diagnosed as having ALRI.
  - Refer every study child who diagnosed as having ALRI, or any other serious childhood diseases based on the Integrated Management of Neonatal and Childhood Illness algorism using the referral form.

# Annex: Parental informed consent form (English-Version)

# Introduction and purpose

Dear participant, my name is ____________________; I work for a study project on prevalence and risk factors of acute lower respiratory infection among children living in households of “Mecha” Health and Demographic Surveillance System Sites, Northwest Ethiopia. The research project is coordinated by the principal investigator Mesafint Molla and fully supported by Bahir Dar University through the “Mecha” Health and Demographic Surveillance (MHDSS) research funding system. The goal of the research project is to investigate the prevalence of childhood acute lower respiratory infection and associated factors in your locality, and we are asking parents/ primary caretakers in the selected clusters/“*Gots*” of “Mecha” district to participate in this study and we would, therefore, like your permission to interview and your participation is completely voluntary.

# Procedures፡

The major procedures for this study are a face-to-face interview with the index child’s mother/ primary caretaker using a structured questionnaire, observation and assessment of a currently sick child to identify childhood ALRI using the Integrated Management of Neonatal and Childhood Illness (IMNCI) algorism.

# Risks

There are no known risks related to the study and none of the procedures will be invasive. The negative aspect of participation will be the visits by trained health workers for data collection which necessitate answering questions about your child’s health and household cooking practices which may be viewed as inconvenient.

# Benefits

No monetary or other compensation will be offered for participation.

# Confidentiality

All study-related information will be stored securely and information collected from the community during the entire course of the study will be maintained strictly confidential. All ALRI case finding results will be kept strictly confidential and childhood ALRI diagnosis activities will be conducted in the participant’s private house, and field workers will never inform the ALRI status to other members of their group.

# Right to refuse or withdraw

Mothers/ primary caretakers are free to refuse participation, withhold information, and skip questions to answer as well as to withdraw from the study at any time without prejudice or coercion.

# Persons to contact

- Principal Investigator’s name: Mesafint Molla at Bahir Dar University, College of Medicine and Health Sciences, Tel: +251918121064/+251928505424.
- Supervisor’s name: Professor Getu Degu, at Bahir Dar University, College of Medicine and Health Sciences, Tel: +251918776010.

# Participants written agreement

I have been well aware of that this research undertaking is a study project on prevalence and risk factors of acute lower respiratory infection among children living in households of “Mecha” Health and Demographic Surveillance System Sites, Northwest Ethiopia which is fully supported and coordinated by Bahir Dar University, College of Medicine and Health Sciences, School of Public Health and the designate principal investigator is Mesafint Molla.

I have been fully informed in the language I understand about the research project objective. I have been also informed that all the information I shall provide to the interviewer will be kept confidential. I also knew that I have the right to withhold information, skip questions to answer or to withdraw from the study at any time. I have acquainted that nobody will impose me to explain the reason for withdrawal. I have assured that the right to ask information that is not clear about the research before and/or during the research work and to contact Bahir Dar University, College of Medicine and Health Sciences IRB Office.

I have read this form, or it has been read to me in the language I comprehend and understood the condition stated above, therefore, I am willing and confirm my participation by signing the consent:

Name of participant giving written consent: ______________________________________,

Signature/figure print: __________.

Name of person obtaining written consent:________________________________________,

Signature: ____________.
